# Creatinine and cystatin C. There is a lack of studies that compare endogenous and exogenous GFR markers in ICU patients

**DOI:** 10.3109/03009734.2011.578763

**Published:** 2011-06-29

**Authors:** Anders Larsson

**Affiliations:** Department of Medical Sciences, Clinical Chemistry, Uppsala University, Uppsala, Sweden

Dear Sir,

We appreciate the letter to the Editor by Dr Chia-Ter Chao regarding our article entitled ‘Significant differences when using creatinine, modification of diet in renal disease, or cystatin C for estimating glomerular filtration rate in ICU patients’ (1).

Kidney function is a very important variable in intensive care patients, and we believe that this variable has not gained sufficient attention. We certainly agree with the statement that ‘both formulae will not suffice as good estimators of renal function in critically ill patients with acute kidney injury’. The problem is that these two markers are widely analyzed in intensive care patients and that the results are often used indiscriminately.

We agree that both markers are subject to interference. However, we would like to question the comment that serum cystatin C level is confounded by inflammation status. Several studies have shown an association between inflammation and serum cystatin C levels, but this may be mediated by kidney damage induced by inflammation. We have in a recently published study shown that inflammation per se had no effect on cystatin C levels (2).

We thank Dr Chia-Ter Chao for bringing up the effect of sampling time on creatinine and cystatin C results (3). This is an important aspect that we failed to mention in our original study. The sampling time may contribute to the differences, but we find the same differences between creatinine and cystatin C-estimated glomerular filtration rate (GFR) also in patients who have spent several days in the intensive care unit (ICU).

The combination of creatinine-based and cystatin C-based results for estimation of GFR is an interesting approach. However, this strategy is probably most effective if there is a fair agreement between the two markers and the mean of the two estimates can be used. The problem in intensive care patients is that the differences between the two estimates are profound and that we do not know which of the markers is correct. We have tried to find comparisons in the literature between creatinine or cystatin C and iohexol or other exogenous GFR markers in intensive care patients. To our surprise, we were not able to find any such studies.

We hope that *UJMS* readers will continue the discussion on the use of endogenous GFR markers. Hopefully, this discussion could also lead to the initiation of studies that compare creatinine/cystatin C with exogenous GFR markers in intensive care patients so that in the future we will know how we should interpret GFR results based on endogenous GFR markers.
